# Health Status of Adult Cynomolgus and Rhesus Monkeys Produced by Embryo Splitting

**DOI:** 10.3390/ani16213459

**Published:** 2026-11-02

**Authors:** Zifan Li, Yitong Yin, Yashi Wu, Yanchao Duan, Zhigang Chen, Yuandong Niu, Nannan Gong, Yu Kang, Yuyu Niu, Chenyang Si

**Affiliations:** 1 State Key Laboratory of Primate Biomedical Research, Institute of Primate Translational Medicine, Kunming University of Science and Technology, Kunming 650500, China; 2 Yunnan Key Laboratory of Primate Biomedical Research, Kunming 650500, China; 3 Southwest United Graduate School, Kunming 650092, China

**Keywords:** nonhuman primates (NHPs), embryo splitting, reproductive performance, health

## Abstract

Animals derived from embryo splitting have been successfully produced in livestock farming, and their milk and meat products have entered the human food market. However, although monkeys have been obtained through the same technique, their adult health status remains poorly studied. Here, we performed a comprehensive and systematic evaluation of five adult cynomolgus and two rhesus monkeys derived from embryo splitting, including hematological and biochemical analyses, glucose metabolism assessment, cardiovascular evaluation, bone mineral density examination, brain MRI, and reproductive performance assessment. No significant differences were found in measured parameters between sp-monkeys and controls in cynomolgus monkeys (5 vs. 4), with similar trends in rhesus monkeys. Because only two rhesus sp-monkeys and three rhesus controls were available, the rhesus data were not statistically compared. These results provide no evidence of obvious adverse effects on adult health and offer preliminary support for the safety of this technique in biomedical research.

## Simple Summary

Embryo splitting is a technique used in livestock to produce genetically identical offspring, and products like milk and meat from such animals are already sold for human consumption. While this method has also been used to create monkeys for research, little is known about the long-term health of these animals as adults. In this study, we thoroughly examined five adult cynomolgus monkeys and two adult rhesus monkeys that were born through embryo splitting. We tested their blood, metabolism, heart function, bone density, brain structure, and ability to reproduce. Compared with age-matched monkeys born via natural methods, we found no meaningful differences in any of the measured health indicators. Our findings suggest that embryo splitting may not harm the adult health of monkeys, and provide preliminary support for the safety of this technique in future biomedical studies.

## 1. Introduction

Life begins with a totipotent fertilized egg. The early embryo (from the cleavage stage to the blastocyst stage) exhibits remarkable developmental plasticity—the ability to self-repair and adjust to external disturbances. Examples include damage compensation (a single blastomere from a two-cell embryo can develop into a complete individual), embryo aggregation (multiple embryos can fuse into one), and artificial splitting (dividing an embryo into two or more equal parts to produce genetically identical twins or multiples) [1]. Embryo splitting technology is based on developmental plasticity. It uses enzymatic treatment and micromanipulation to divide an embryo at the cleavage or blastocyst stage into two or more equal parts, thereby generating monozygotic twins or multiple offspring.

This technique has been successfully applied in the livestock industry to promote the propagation of superior livestock (for meat, milk or both) and has resulted in the production of monozygotic twins or multiple births of sheep [2,3,4], pigs [5], horses [6] and cattle [7,8]. Particularly in cattle, bisected embryos achieve pregnancy rates comparable to those of intact embryos (64.2–74.6%), with monozygotic twinning rates of up to 66.6% [9,10]. Moreover, because monozygotic twins can multiply elite genotypes and accelerate genetic gain, embryo splitting has become an important tool in animal breeding [11,12]. For example, optimized blastomere separation methods have achieved pregnancy rates approaching 79% [7]. Meat and milk from these animals are no different from those derived from naturally conceived animals and they have entered the human food market [13,14,15]. These findings suggest that embryo splitting could be very useful for breeding valuable farm animals. Non-human primates (NHPs) are very similar to humans in genetics, body structure, physiology, immunity, and reproduction, so they are important models in medical research, drug development, and testing before human trials [16,17]. However, they reproduce slowly and have long reproductive cycles, which limits this kind of research. Therefore, more studies are needed to see whether this technique can also help NHPs reproduce more effectively while keeping their offspring healthy and safe in the long term.

So far, the application of embryo splitting in NHPs has been relatively limited. Previous studies only showed no differences in gestational cycle and birth weight between offspring and controls [18,19]. By optimizing embryo splitting techniques, we generated six cynomolgus monkeys and four rhesus monkeys from split embryos, and, for the first time, successfully derived healthy live offspring together with genetically matched autologous embryonic stem cell lines from a single embryo. Although we observed that these monkeys maintained normal growth in weight and height without abnormalities over the course of one year [20], the long-term health of these animals in adulthood has not been thoroughly assessed.

In this study, we performed a comprehensive assessment of seven 6-year-old monkeys derived from embryo splitting (sp-monkeys) and seven same-aged controls, including hematological and biochemical analyses, glucose tolerance testing, cardiac and skeletal evaluations, as well as brain magnetic resonance imaging (MRI) and reproductive capacity assessment. Although minor differences were observed in certain parameters, all values remained within the normal reference range. In cynomolgus monkeys, no measured parameter differed significantly from controls; rhesus data were not statistically compared because of the small sample size. Collectively, we found no evidence of deleterious long-term effects of embryo splitting on adult monkeys in this cohort, although the small sample size limits definitive conclusions.

## 2. Materials and Methods

### 2.1. Animals

Monkeys derived via embryo splitting are hereafter referred to as sp-monkeys. A total of nine cynomolgus monkeys (5 sp-monkeys and 4 controls) and five rhesus monkeys (2 sp-monkeys and 3 controls) were included in this study. These sp-monkeys were generated by embryo splitting in a previous study [20], whereas the control animals were naturally conceived. All animals were housed at the Primate Translational Medicine Research Institute, Kunming University of Science and Technology. The animal facility is accredited by AAALAC International. Animals produced by embryo splitting and naturally conceived animals were housed under identical conditions and provided with the same food and care during the first six years of life. Briefly, before weaning, the animals were either reared by their mothers or housed one or two per infant incubator. After weaning, they were group-housed with animals of the same sex, with approximately 35 animals typically housed in each group-housing unit. During the assessment period of this study, the animals were transferred to individual cages measuring 80 cm × 80 cm × 80 cm and were returned to the group-housing environment three months later. No special care or husbandry procedures were provided to the sp-monkeys. Animals were fed a commercial non-human primate maintenance diet (manufactured by Kunming Yaling Biotechnology Co., Ltd., Kunming, China, in accordance with GB 14924.3-2010, Laboratory animals—Nutrients of formula feeds, Standards Press of China, Beijing, China, 2010) twice daily (50–100 g per feeding). In the afternoon, they received a limited supplement of fruit, vegetables, or a mixed grain diet, with water available ad libitum. All experimental procedures were reviewed and approved by the Institutional Animal Care and Use Committee of Kunming University of Science and Technology (approval number KUST202301008).

### 2.2. Blood Sample Collection

Blood samples were collected when the animals reached six years of age. Blood samples were collected from non-anesthetized animals. All monkeys were fasted overnight for 12 h before blood collection, with free access to water. Blood samples were obtained from a hindlimb vein of each unanesthetized animal. Hair at the venipuncture site was shaved and the skin was disinfected with 75% ethanol. Blood was then collected using a disposable venous blood collection needle (0.55 × 19 mm; Shandong Aosaite Medical Devices Co., Ltd., Heze, China) into plain vacuum blood collection tubes (Hebei Kangweishi Medical Technology Co., Ltd., Shijiazhuang, China), with 4 mL obtained from each animal.

### 2.3. Hematological Analysis

Hematological analyses were performed using a Sysmex XN-1000V automated hematology analyzer (Sysmex Corporation, Kobe, Japan). Briefly, approximately 1 mL of whole blood was collected into EDTA-K2-anticoagulated vacuum tubes (Hebei Kangweishi Medical Technology Co., Ltd., Shijiazhuang, Hebei, China) and analyzed within 2 h of collection. Before analysis, samples were gently inverted several times to ensure thorough mixing. The hematologic parameters included: red blood cell count (RBC, 10^12^/L), hemoglobin (HGB, g/dL), hematocrit (HCT, %), mean corpuscular volume (MCV, fL), mean corpuscular hemoglobin mass (MCH, pg), mean corpuscular hemoglobin concentration (MCHC, g/L), platelets (PLT, 10^9^/L), red cell distribution width-standard deviation (RDW-SD, fL), red blood cell distribution width-coefficient of variation (RDW-SV, %), mean platelet volume (MPV, fL), platelet distribution width (PDW, fL), platelet large cell ratio (P-LCR, %), plateletcrit (PCT, %), neutrophils (NEUT, 10^9^/L), lymphocytes (LYMPH, 10^9^/L), monocytes (MONO, 10^9^/L), eosinophils (EO, 10^9^/L), basophils (BASO, 10^9^/L), neutrophil percentage (NEUT%, %), lymphocyte percentage (LYMPH%, %), monocyte percentage (MONO%, %), eosinophil percentage (EO%, %), and basophil percentage (BASO%, %), white blood cell count (WBC, 10^9^/L).

### 2.4. Biochemical Analysis

For serum biochemistry, 4 mL of non-anticoagulated whole blood was centrifuged at 4 °C for 10 min at 3000 rpm, and the supernatant (serum) was collected. Serum samples were analyzed using an automated biochemical analyzer (Cobas 311, Roche Diagnostics, Mannheim, Germany). The serum chemistry parameters included: alanine aminotransferase (ALT, U/L), aspartate aminotransferase (AST, U/L), alkaline phosphatase (ALP, U/L), creatine kinase (CK, U/L), total bilirubin (T-BIL, µM), direct bilirubin (D-BIL, µM), total protein (TP, g/L), albumin (ALB, g/L), globulin (GLB, g/L), and albumin/globulin Ratio (A/G), blood urea (UREA, mM), creatinine (CREA, μM), serum uric acid (UA, μM), high-density-lipoprotein cholesterol (HDL-CH, mmol/L), low-density-lipoprotein cholesterol (LDL-CH, mmol/L), total cholesterol (TC, mmol/L), triglyceride (TG, mmol/L), apolipoprotein A1 (APOA1, g/L), apolipoprotein B (APOB, g/L), creatine kinase (CK, U/L), lactate dehydrogenase (LDH, U/L), calcium (Ca, mM), and phosphorus (P, mM).

### 2.5. Intravenous Glucose Tolerance Test (IVGTT)

One week after the hematological examination, IVGTT was performed in unanesthetized animals by experienced technicians. After an overnight fast, 0.5 g/kg of 50% glucose was infused i.v. into the monkeys within 1 min. Blood glucose concentrations were measured with a handheld glucometer at 0, 30, 60, 90 and 120 min after injection. Insulin and C-peptide levels at the same time points were measured by Roche Cobas e 411 electrochemiluminescence analyzer (Roche Diagnostics GmbH, Mannheim, Germany).

### 2.6. Echocardiography and Electrocardiography

Echocardiography and bone mineral density measurements were performed one week after the IVGTT. Monkeys were anesthetized with ketamine (10 mg/kg, intramuscular injection) and positioned in the supine or left lateral decubitus position on a temperature-controlled platform. Measurements were performed in accordance with previously described methods and terminology [21], and included the following parameters: aortic sinus diameter (AoS), ascending aortic diameter (AA), left atrial diameter (LAD), right ventricular diameter (RVD), interventricular septal thickness (IVS), left ventricular posterior wall thickness (LVPW), left ventricular end-diastolic diameter (LVID), left ventricular end-systolic diameter (LVIDS), left ventricular end-diastolic volume (EDV), stroke volume (SV), ejection fraction (EF), fractional shortening (FS), right atrial transverse diameter (RA-T), right atrial longitudinal diameter (RA-L), mitral inflow velocities E/A ratio, tissue Doppler imaging (TDI) mitral annular e′/a′ ratio at the septal annulus, TDI mitral annular e′/a′ ratio at the lateral annulus, and peak aortic valve outflow velocity (AV). A 12-lead electrocardiogram was recorded using a fully digital multichannel ECG system (BeneHeart R12, Mindray, Shenzhen, China).

### 2.7. Bone Mineral Density Assessment

Monkeys were anesthetized with ketamine (10 mg/kg, intramuscular injection) and whole-body dual-energy X-ray absorptiometry (DXA) scans were acquired in the anteroposterior (AP) projection using a GE Lunar iDXA densitometer (GE Healthcare, Chicago, IL, USA). Whole-body composition was subsequently analyzed using enCORE software (version 18).

### 2.8. MRI Acquisition

MRI was performed one week after echocardiography and bone mineral density measurements. The MRI data were collected using the Siemens 3T Prisma MRI scanner (Siemens, Erlangen, Germany) and acquired with a scan time of approximately 50 min per animal. Before the scanning procedure, the monkeys were anesthetized with intramuscular Zoletil (tiletamine + zolazepam) (3 mg/kg) (Virbac, Carros, France) and Sumianxin (0.5 mg/kg) (Dunhua Shengda Animal Medicine Co., Ltd., Dunhua, China). 3D T1w images were acquired using the following parameters: TR = 1400 ms, TE = 3.55 ms, inversion time = 900 ms, flip angle = 8 degrees, FOV read = 135 mm, slice thickness = 0.6 mm, voxel size = 0.6 mm × 0.6 mm × 0.6 mm, slices per slab = 160. The T2w images were collected with the following parameters: TR/TE = 2500/520 ms, slice thickness = 0.6 mm, voxel size = 0.6 mm × 0.6 mm × 0.6 mm, slices per slab = 128, FOV read = 115 mm.

### 2.9. Structural MRI Data Preprocessing

Using the DAPBI toolbox on the MATLAB (version R2021b) platform, all NIfTI-format (.nii) images of gray matter volume (VBM) and white matter volume (WMV) from the participants were processed. First, the ROI Signal Extractor module in DAPBI was used to extract volumetric values from predefined regions of interest (ROIs), thereby generating subject-specific VBM and WMV measures.

### 2.10. Oocyte Collection, ICSI, Embryo Culture, and Embryo Transplantation

Embryo collection and transfer were performed as described previously [20]. Briefly, superovulation was initiated on days 1–3 of the menstrual cycle. rhFSH (recombinant human follitropin alfa, GONAL-F, Merck Serono, Darmstadt, Germany) was administered intramuscularly for 8 consecutive days, followed by rhCG (recombinant human chorionic gonadotropin alfa, OVIDREL, Merck Serono) on day 9. Oocytes were collected by laparoscopic follicular aspiration 32–35 h after rhCG administration. For oocyte collection, the monkeys were anesthetized with ketamine (10 mg/kg body weight, intramuscular injection). To evaluate the reproductive function of oocytes and sperm from sp-monkeys, we performed superovulation in four cynomolgus sp-monkeys and two control cynomolgus monkeys. MII oocytes were subjected to intracytoplasmic sperm injection (ICSI), and fertilization was confirmed by the presence of two pronuclei. Zygotes were cultured in chemically defined, protein-free hamster embryo culture medium-9 (HECM-9) supplemented with 10% fetal bovine serum (Hyclone Laboratories, Logan, UT, USA, SH30088.02) at 37 °C in 5% CO_2_. High-quality embryos at the morula to blastocyst stage were transferred into surrogate monkeys.

### 2.11. Semen Collection and Sperm Functional Assessments

Male monkeys were anesthetized by intramuscular injection of ketamine and positioned supine on the procedure table. Their scrotums and penises were washed with physiological saline. Aluminum foil electrodes wrapped in absorbent cotton were then placed at both ends of the penis, and semen was collected by a multipurpose physiopharmacological stimulator (JL-D, Shanghai Precision Instruments Co., Ltd., Shanghai, China) through intermittent stimulation.

Sperm functional assessments included two cynomolgus sp-monkeys and three control cynomolgus monkeys. One control cynomolgus monkey also completed all other assessments; the other two were used for functional assessments only. Sperm motility, acrosome integrity, DNA integrity and mitochondrial membrane potential were assessed according to previously reported methods [22]. Briefly, the sperm were diluted to an appropriate concentration of approximately 1 × 10^6^/mL and examined motility under a microscope using a hemocytometer (BRAND, 717810, Wertheim, Germany). Sperm integrity was evaluated using FITC-conjugated peanut agglutinin (PNA-FITC) (Sigma-Aldrich, L7381, St. Louis, MO, USA), 10 μL of the sperm suspension was spread evenly onto a glass slide and fixed with paraformaldehyde (PFA) for 15 min. The slide was then incubated with 50 μL of PNA-FITC solution (10 μg/mL) in the dark at 37 °C for 30 min, followed by examination under a fluorescence microscope. Sperm displaying uniform green fluorescence had intact acrosomes, whereas those showing little or no green fluorescence were classified as acrosomal sperm impairment. Mitochondrial membrane potential was evaluated using a JC-1 assay kit (Solarbio, M8650, Beijing, China).

### 2.12. Statistical Analysis

Data are presented as the mean ± SD. All analyses were exploratory and were performed using SPSS version 27 or GraphPad Prism 10. For normally distributed variables, differences between groups were compared using an independent-samples *t*-test. For variables that did not meet the assumption of normality in at least one group, the Mann–Whitney U test was used for between-group comparisons. Given the exploratory nature of the study and the small sample size, no adjustment for multiple comparisons was applied. However, the resulting *p* values should be interpreted cautiously. A two-sided *p* < 0.05 was considered statistically significant. Non-significant differences were not interpreted as evidence of biological equivalence.

## 3. Results

### 3.1. Sp-Monkeys Show Normal Growth

**Figure 1:**
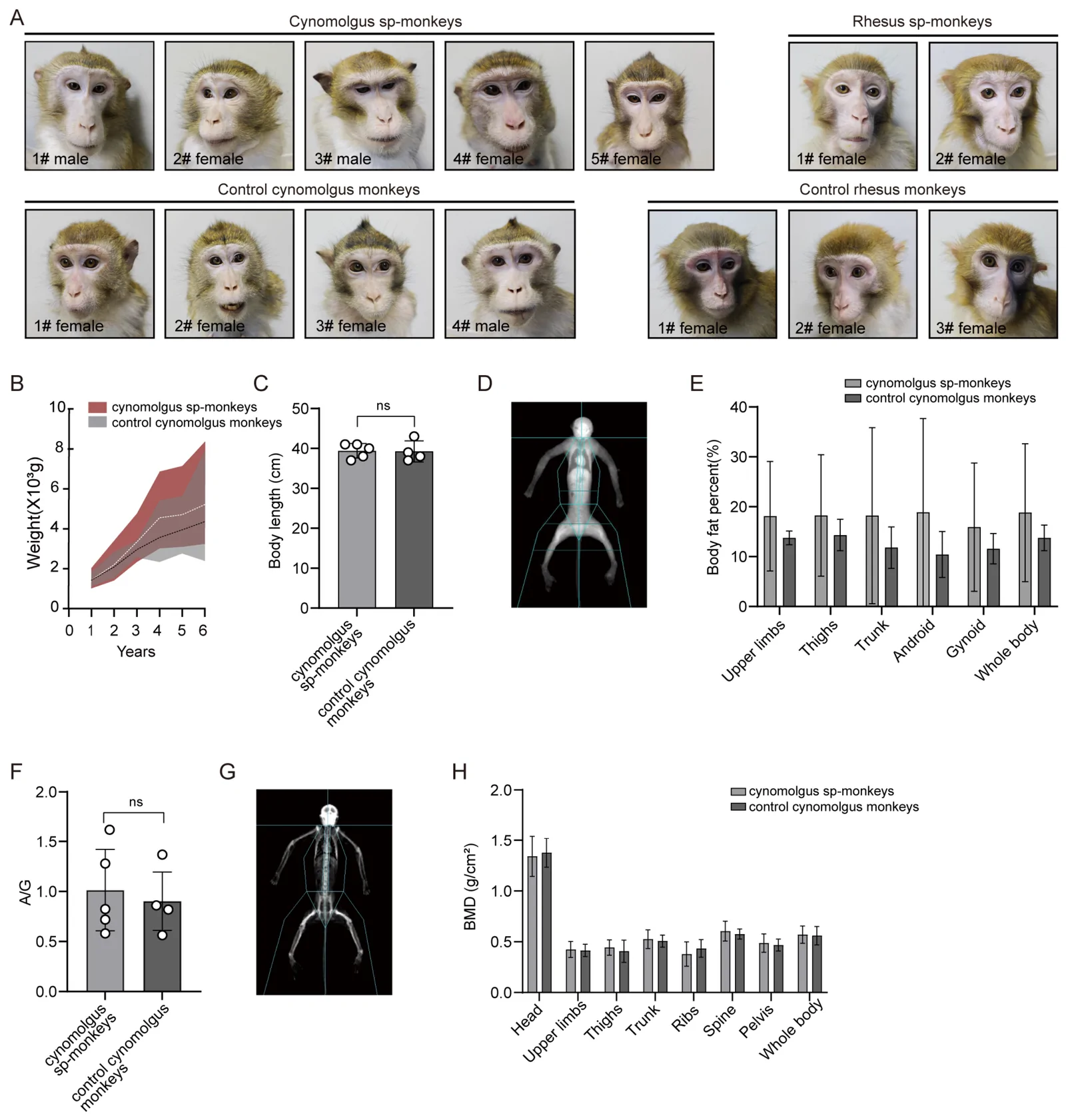
Body condition assessment of cynomolgus sp-monkey and control groups. (**A**) Representative images of cynomolgus sp-monkeys and controls. (**B**) Body weight changes in cynomolgus sp-monkeys and controls from 1 to 6 years of age. White and black dashed lines indicate the mean values of sp-monkeys and controls, respectively. The shaded area represents the 95% confidence interval, reflecting the variability of the data. The upper and lower edges of the shaded area represent the maxima and minima, respectively. The statistical analysis utilized multiple two-tailed *t*-tests without adjustment. (**C**) Comparison of body length at 6 years of age between cynomolgus sp-monkeys and controls, ns, not significant (*p* > 0.05). (**D**) Representative DXA images of whole-body composition. (**E**) Quantitative comparisons of body composition in the upper limbs, thighs, trunk, android, gynoid, and whole body. (**F**) Statistical analysis of the android-to-gynoid (A/G) ratio in the cynomolgus sp-monkeys and controls. (**G**) Representative DXA images of bone mineral density (BMD). (**H**) Comparisons of bone mineral density in the upper limbs, lower limbs, trunk, ribs, spine, pelvis, and whole body between sp-monkeys and controls. Data are presented as mean ± SD. (cynomolgus sp-monkeys, *n* = 5; controls, *n* = 4). The statistical analysis utilized the unpaired two-tailed Student’s *t* test.

In this study, we compared six-year-old sp-monkeys with controls. The sp-monkeys consisted of five cynomolgus (three females, two males) and two female rhesus monkeys, while the controls included four cynomolgus (three females, one male) and three female rhesus monkeys (Figure 1A and Figure S1A). Over six years, we measured body weight each year and observed no difference between cynomolgus sp-monkeys and control cynomolgus monkeys (Figure 1B). At six years of age, the cynomolgus sp-monkeys also reached an adult body length of approximately 40 cm, comparable to that of the controls (Figure 1C). To assess body composition, we further performed body fat analysis to evaluate whether embryo splitting affects adipose tissue deposition, which is a critical indicator of metabolic health under the same housing conditions [23]. We found no difference between the two groups in fat content across body regions and Android/Gynoid (A/G) ratios (Figure 1D–F), a metric for osteoporosis, metabolic syndrome, and cardiovascular risk [24,25]. Although the difference in body fat percentage between the two groups was not statistically significant, sp-monkeys showed a numerically higher mean body fat. Inspection of individual data indicated that this trend was largely attributable to two sp-monkeys, one female and one male, with relatively high adiposity; the remaining sp-monkeys had values comparable to those of naturally conceived controls. Beyond fat distribution, bone mineralization is another key measure of developmental quality. We therefore used DXA to assess bone mineral density (BMD) in cynomolgus sp-monkeys. The results showed that the BMD content of the head, upper limbs, thighs, trunk, ribs, spine, pelvis and whole body of cynomolgus sp-monkeys was consistent with controls (Figure 1G,H). In addition, we also observed that the growth and development, fat distribution, and BMD of rhesus sp-monkeys were similar to those of the controls (Figure S1B–E).

Overall, when analyzed separately by species, the present data reveal no significant intergroup differences in body weight, height, adiposity, or bone mineral density in cynomolgus monkeys, with similar trends in rhesus monkeys, suggesting that embryo splitting may not adversely affect overall growth or skeletal development, although the small sample size limits definitive conclusions.

### 3.2. Minor Alterations in Blood Parameters and Glucose Dynamics in Sp-Monkeys

Although the two groups were similar in body weight, height, fat distribution, and A/G ratio, these external parameters do not fully capture internal metabolic or organ function. We therefore performed blood biochemical analysis to comprehensively assess the overall health status of sp-monkeys. Most hematological indices did not differ significantly from those of the controls. However, PDW and WBC showed significant differences, and LYMPH exhibited a more pronounced difference (Table 1). These differences may possibly reflect individual variation or sampling factors, rather than a real effect of embryo splitting. However, this remains a possible explanation and cannot be established from the present data. Notably, no significant differences were found between sp-monkeys and controls for any of the measured biochemical parameters (Table 2).

**Figure 2:**
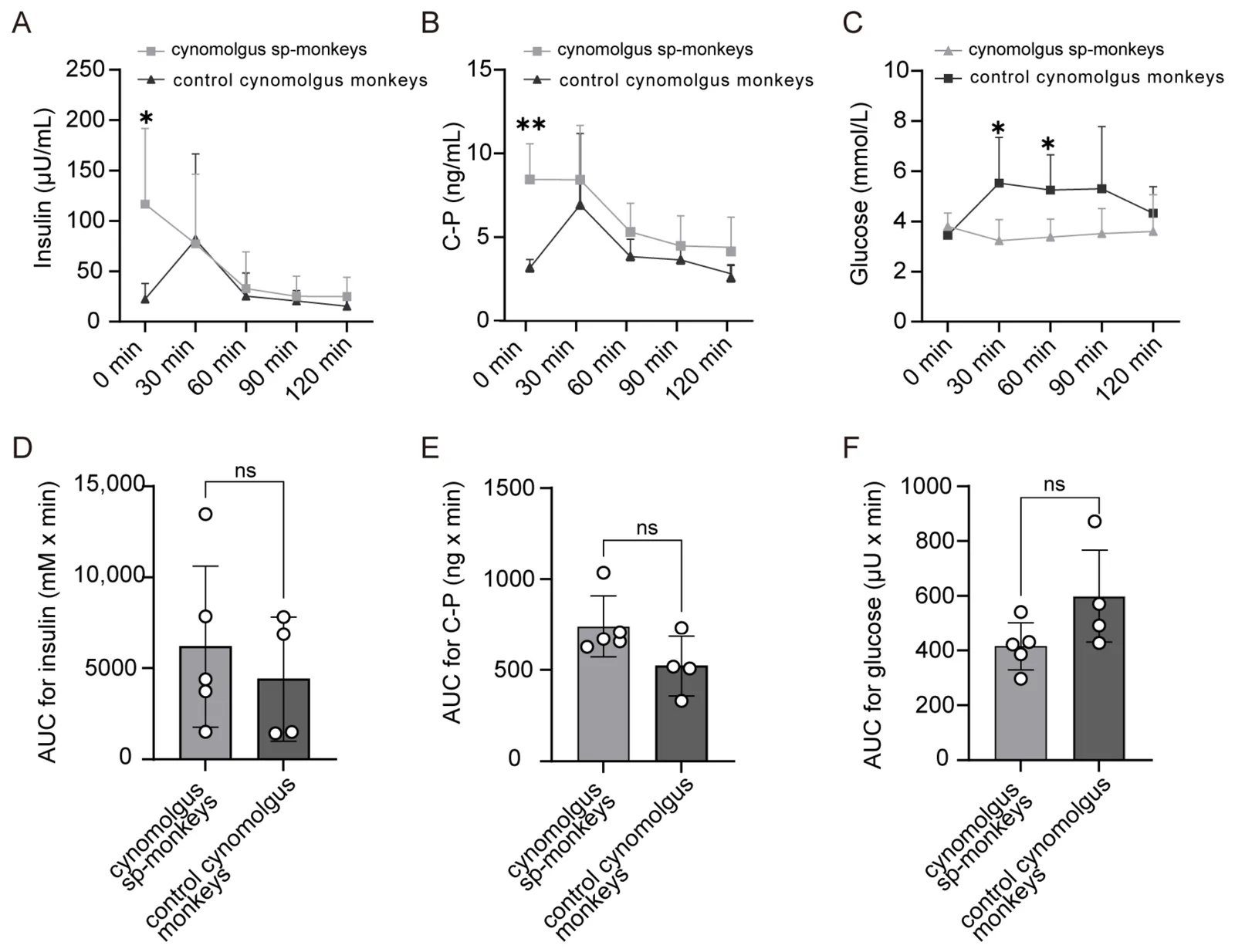
Assessment of glucose metabolism in cynomolgus sp-monkeys by intravenous glucose tolerance test (IVGTT). (**A**–**C**) Changes in insulin levels (**A**), c-peptide release (**B**) and glucose levels (**C**) upon IVGTT administered to the cynomolgus sp-monkeys and controls; (**D**–**F**) AUC analysis of insulin secretion (**D**), C-peptide release (**E**), and blood glucose levels (**F**) during IVGTT in cynomolgus sp-monkeys and controls. Data are presented as mean ± SD. (cynomolgus sp-monkeys, *n* = 5; controls, *n* = 4). Normally distributed data were analyzed by independent samples *t* test, and non-normally distributed data by non-parametric tests. * *p* < 0.05, ** *p* < 0.01; ns, not significant.

To assess glucose homeostasis and pancreatic β-cell function, we performed intravenous glucose tolerance tests (IVGTT) on cynomolgus sp-monkeys and control cynomolgus monkeys. At baseline, the cynomolgus sp-monkeys showed significantly higher insulin and C-peptide levels than the control cynomolgus monkeys (Figure 2A,B), whereas fasting blood glucose did not differ significantly between groups (Figure 2C). After glucose challenge, blood glucose levels were significantly lower in cynomolgus sp-monkeys than in the control cynomolgus monkeys at 30 and 60 min, and returned to near baseline in both groups by 120 min (Figure 2C). Although the area under the curve (AUC) for glucose was numerically lower in the cynomolgus sp-monkeys, whereas the AUCs for insulin and C-peptide were numerically higher, none of the between-group differences were statistically significant (Figure 2D–F). These findings indicate that cynomolgus sp-monkeys exhibited elevated basal islet activity and accelerated early glucose clearance, but normal overall glucose tolerance, implying that embryo splitting may affect glucose dynamics primarily via basal and early-phase insulin secretion.

In summary, sp-monkeys showed comparable hematological and biochemical profiles to controls, except for minor blood cell differences possibly attributable to individual or procedural variation. However, in cynomolgus monkeys, sp-monkeys exhibited enhanced basal islet activity and faster early glucose clearance, indicating that embryo splitting may subtly modulate glucose dynamics without affecting overall metabolic health.

### 3.3. No Detectable Cardiac and Brain Abnormalities in Sp-Monkeys

**Figure 3:**
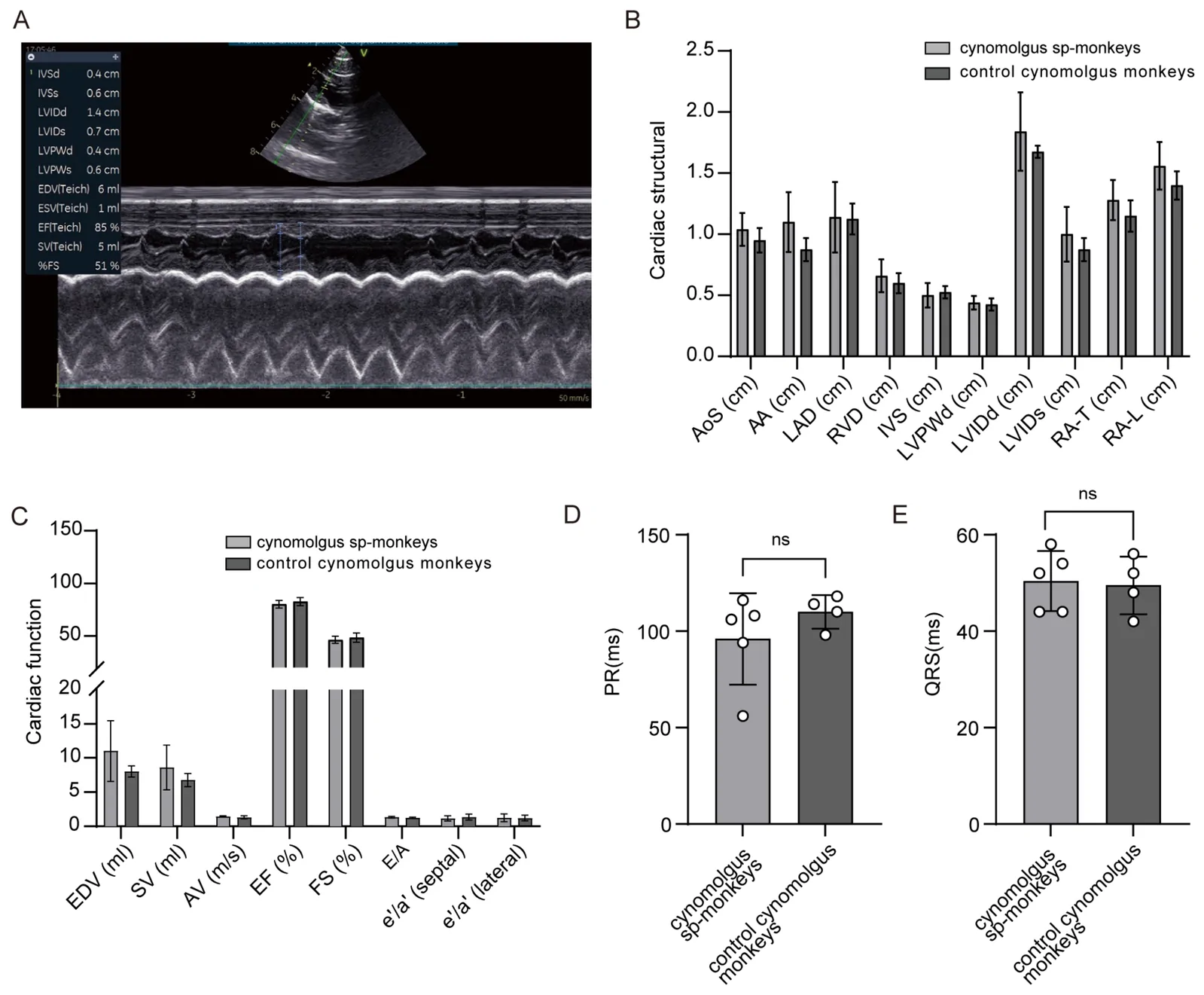
Comparison of cardiac structure and function between cynomolgus sp-monkeys and controls. (**A**) Representative ultrasound images of left ventricular size and function. (**B**) Quantitative comparisons of cardiac structural parameters. AoS, aortic sinus diameter; AA, ascending aortic diameter; LAD, left atrial diameter; RVD, right ventricular diameter; IVS, interventricular septal thickness; LVPWd, left ventricular posterior wall thickness; LVIDd, left ventricular end-diastolic diameter; LVIDs, left ventricular end-systolic diameter; RA-T, right atrial transverse diameter; RA-L, right atrial longitudinal diameter. (**C**) Quantitative comparisons of cardiac functional parameters. E/A, mitral inflow velocities E/A ratio; e′/a′ (septal), tissue Doppler imaging (TDI) mitral annular e′/a′ ratio at the septal annulus; e′/a′ (lateral), TDI mitral annular e′/a′ ratio at the lateral annulus; EDV, left ventricular end-diastolic volume; SV, stroke volume; EF, ejection fraction; FS, fractional shortening; AV, peak aortic valve outflow velocity. (**D**,**E**) Quantitative comparison of the PR interval (**D**) and QRS duration (**E**). Data are presented as mean ± SD. (cynomolgus sp-monkeys, *n* = 5; controls, *n* = 4). The statistical analysis utilized the unpaired two-tailed Student’s *t* test. ns. not significant.

Because peripheral blood and glycemic indices only indirectly reflect cardiovascular status, we further used echocardiography to comprehensively evaluate potential abnormalities in cardiac structure and function. This analysis was performed in both cynomolgus and rhesus monkeys, and the data were analyzed separately for each species. No significant differences were observed between sp-monkeys and controls in the dimensions of cardiac structures (chamber sizes: LA, RV, RA-T, RA-L, LVIDd, LVIDs; wall thicknesses: IVSd, LVPWd; and great vessel diameters: AoS, AA) (Figure 3A,B and Figure S2A). Likewise, functional indices—including diastolic function (E/A; septal and lateral e′/a′ by TDI), systolic function (EDV, SV, EF, FS), and aortic flow (peak AV) were comparable between the two groups (Figure 3C and Figure S2B). To further evaluate cardiac electrical activity and myocardial integrity, we also measured electrocardiographic parameters (PR interval, QRS duration). Similar to the echocardiographic findings, these indices showed no significant differences between the two groups (Figure 3D,E and Figure S2C,D). These findings suggest that embryo splitting has no evident adverse effects on cardiac structure, function, or electrical activity, as assessed by both echocardiography and electrocardiography.

**Figure 4:**
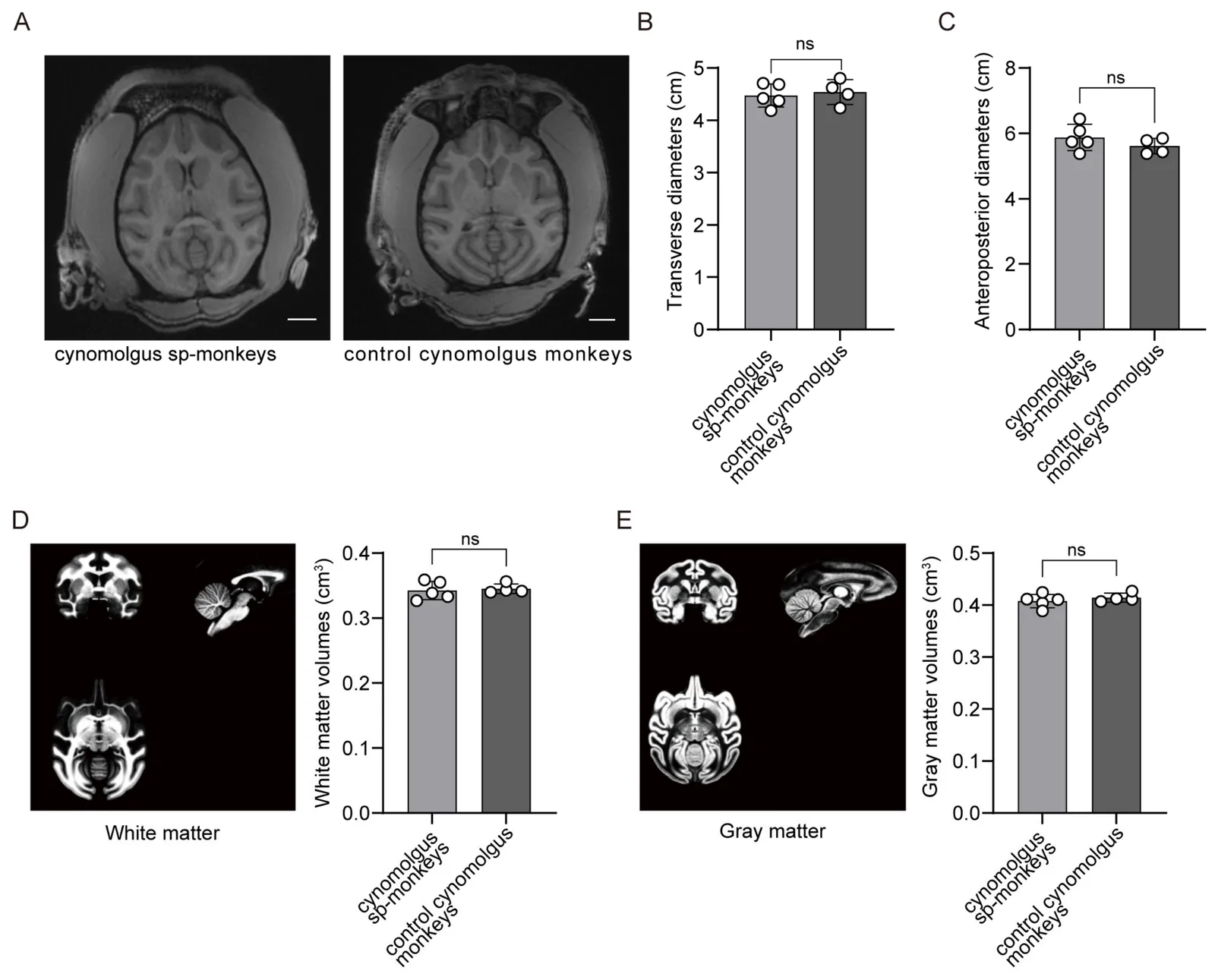
Comparison of brain structure between cynomolgus sp-monkeys and controls. (**A**) Representative MRI scan images of the cynomolgus sp-monkeys and controls; (**B**,**C**) Comparison of brain transverse (**B**) and anteroposterior (**C**) diameters based on T1-weighted MRI images; (**D**,**E**) Atlas of white matter (**D**) and gray matter (**E**) regions constructed from T1- and T2-weighted images, with volumetric comparisons of corresponding regions. Data are presented as mean ± SD (cynomolgus sp-monkeys, *n* = 5; controls, *n* = 4). The statistical analysis utilized the unpaired two-tailed Student’s *t*-test. ns, not significant (*p* > 0.05).

Magnetic resonance imaging (MRI) is a key noninvasive tool for investigating brain development in vivo and has been applied in NHPs to studies of brain development [26,27]. We therefore performed brain MRI in sp-monkeys and controls and found that brain length (anteroposterior diameter) and width (mediolateral diameter) were comparable between the two groups (Figure 4A–C). To further assess potential regional differences, we conducted atlas-based analysis and observed no significant intergroup differences in either white or gray matter regions (Figure 4D,E and Figure S2E,F, Supplementary Video S1). These findings suggest that embryo splitting may not adversely affect gross brain structure or regional tissue volumes.

Collectively, no significant cardiac or brain abnormalities were detected in sp-monkeys. Echocardiography, electrocardiography, and brain MRI all showed comparable parameters between the two groups, suggesting that embryo splitting may not adversely affect heart or brain development.

### 3.4. Assessment of Reproductive Capacity in Sp-Monkeys

**Figure 5:**
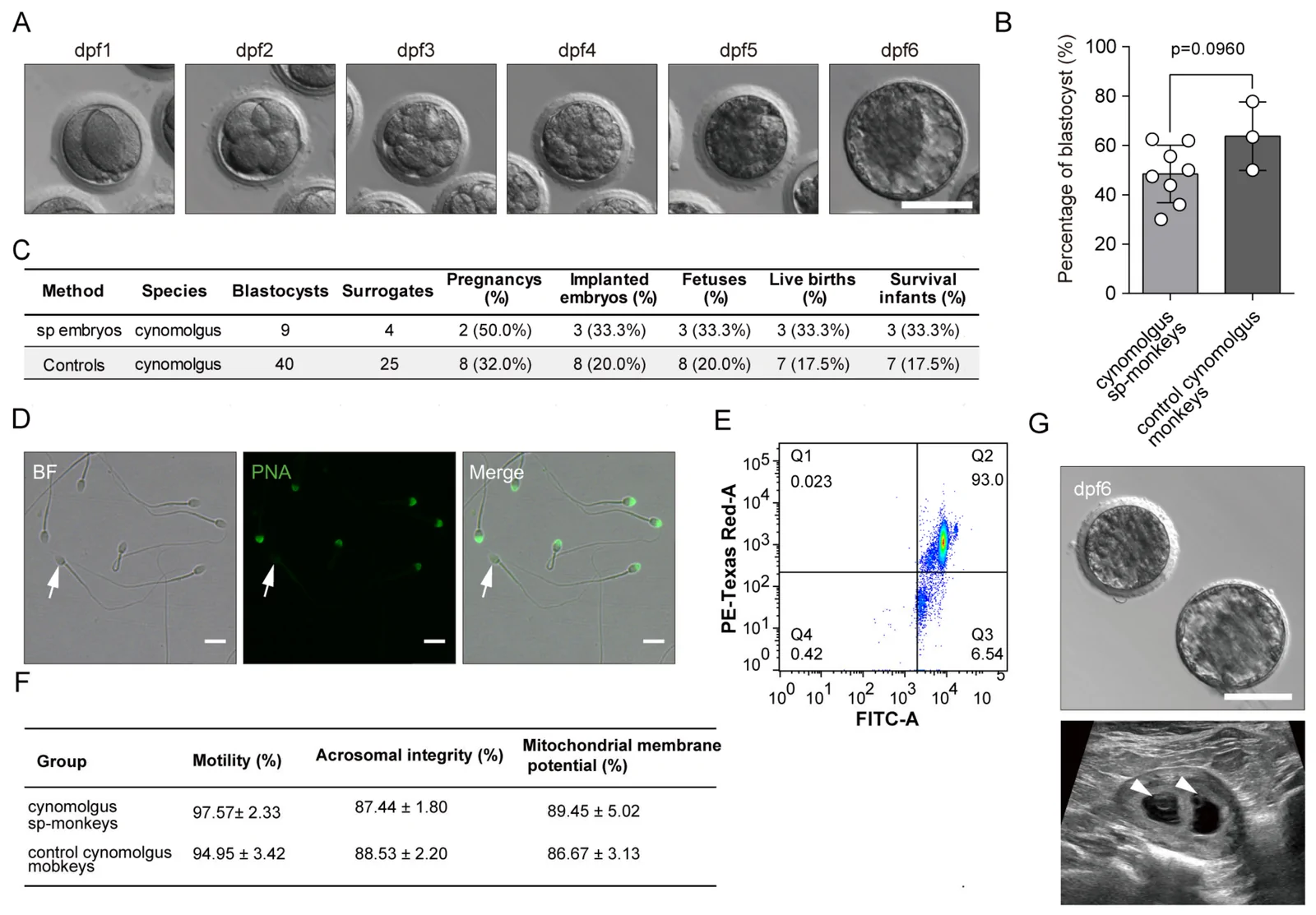
Reproductive performance assessment of cynomolgus sp-monkeys. (**A**) Representative bright-field images of embryos from cynomolgus sp-monkeys at 1 to 6 days post-fertilization (dpf1-dpf6). Scale bar, 100 μm. (**B**) Comparison of blastocyst formation rates between embryos from cynomolgus sp-monkeys (28 embryos from three independent oocyte donors) and the controls (126 embryos from eight independent oocyte donors). Biological replicates were independent oocyte donors. For each donor, the blastocyst formation rate was calculated and donor-level rates were averaged. Bars represent mean ± SEM, unpaired two-tailed Student’s *t*-test. (**C**) Post implantation development of embryos from cynomolgus sp-monkeys and controls. Nine embryos from three cynomolgus sp-monkeys were transferred into four recipients. The control data were obtained from Reference [20]. (**D**) Representative immunofluorescence staining of acrosomes in spermatozoa from two cynomolgus sp-monkeys. Arrows indicate acrosome-damaged sperm. Scale bar, 10 μm. (**E**) Representative flow cytometric analysis of mitochondrial membrane potential in spermatozoa from cynomolgus sp-monkey 1#. Similar results were observed in the other cynomolgus sp-monkey and three controls. (**F**) Statistical analysis of sperm motility, acrosome integrity and mitochondrial membrane potential was performed using individual male cynomolgus monkeys as biological replicates: two cynomolgus sp-monkeys and three controls. Data are presented as group means derived from these monkeys. For each male, five independent measurements were performed and averaged before group comparison. (**G**) Two blastocyst (top panel) were transferred into a female cynomolgus monkey surrogates, with an ultrasound image of the surrogate uterus on day 25 of gestation. The arrow indicates the yolk sac. Scale bar, 100 μm.

To evaluate the effect of embryo splitting on gamete quality, we assessed oocyte and sperm functionality in sp-monkeys. For oocytes, no differences were detected in the number and morphology of mature oocytes (Figure S3A,B). After intracytoplasmic sperm injection (ICSI) of mature oocytes from cynomolgus and rhesus sp-monkeys with control sperm, fertilization and blastocyst rates were comparable to those of control embryos. The blastocysts exhibited distinct inner cell masses, and the developmental timing was consistent with that of control embryos (Figure 5A,B). These findings indicate that oocytes derived from cynomolgus and rhesus sp-monkeys possess normal fertilization capacity and embryonic developmental competence. To further assess the in vivo developmental potential of these embryos, we transferred a total of nine cynomolgus monkey embryos at dpf6 to four female surrogates. After 25 days, we confirmed two pregnancies (one singleton fetus and one twin pregnancy) by ultrasound examination in four cynomolgus monkey surrogates. Implantation rate, pregnancy rate, and live birth rate were subsequently compared between cynomolgus sp-monkeys and controls (Figure 5C). For sperm, we analyzed motility, acrosomal integrity and Mitochondrial membrane potential. No significant differences were observed between cynomolgus sp-monkeys and controls in any of the parameters measured (Figure 5D–F and Figure S3C). We then performed ICSI using sperm from two cynomolgus sp-monkeys and 15 mature oocytes from controls. Of these, 14 mature oocytes were successfully fertilized, and six embryos developed to the blastocyst stage at dpf6. A total of four blastocysts were transferred to two female cynomolgus monkey surrogates, resulting in one pregnancy (with two fetuses) (Figure 5G). The offspring were successfully delivered.

Altogether, these findings preliminarily demonstrate that both oocytes and sperm derived from cynomolgus sp-monkeys are functionally normal. However, both gametes were not always derived from sp-monkeys, no male rhesus sp-monkeys were available, and pregnancy establishment and maintenance were demonstrated in surrogate females. Therefore, these results provide preliminary evidence of gamete functionality and embryonic developmental competence rather than definitive confirmation of normal fertility.

## 4. Discussion

This study systematically evaluated health status of seven adult monkeys (6 years old) generated by embryo splitting. We examined the growth, body composition, BMD, hematology, glucose metabolism, cardiovascular, neurological and reproductive functions of sp-monkeys and found no evidence that embryo splitting adversely affects most physiological parameters, with the notable exception of subtle alterations in blood parameters and glucose dynamics. These results suggest that monkeys derived from embryo splitting were broadly similar to control monkeys across multiple measures, providing preliminary support for the safety of embryo splitting in NPHs, consistent with previously reported findings [19,20,28]. This is further supported by a case report of pregnancy from a 4-cell human embryo with only one surviving blastomere after thawing [29].

The most notable finding of this study was a subtle change in glucose metabolism in cynomolgus sp-monkeys. At baseline, cynomolgus sp-monkeys had significantly higher insulin and C-peptide levels with normal fasting glucose, indicating enhanced basal β-cell activity. After glucose challenge, blood glucose was significantly lower at 30 and 60 min, showing faster early-phase clearance. However, AUC values for glucose, insulin, and C-peptide did not differ between groups, indicating normal overall glucose tolerance. This pattern—elevated basal secretion, faster early clearance, but normal overall tolerance—suggests that embryo splitting may alter the set point of islet function without impairing total glucose disposal. Notably, enhanced function without overall impairment has also been reported in ART-derived monkeys: offspring produced by embryo splitting and ICSI showed faster development in certain cognitive tasks despite no global delays [28]. This echoes our observation of enhanced glucose dynamics with normal overall tolerance. Importantly, all values remained within normal ranges, and no animal developed hyperglycemia or impaired glucose tolerance. These changes likely reflect metabolic plasticity rather than dysfunction.

In contrast, no abnormalities were detected in growth, body composition, BMD, blood biochemistry, cardiovascular, or neurological systems of sp-monkeys from cynomolgus and rhesus monkeys. Body weight, height, fat distribution, and A/G ratio were comparable between sp-monkeys and controls in both species. Minor differences in PDW, WBC, and LYMPH were observed, but they were small, not accompanied by clinical abnormalities or other consistent hematological changes, and were likely due to individual variation or sampling factors rather than embryo splitting. The small sample size limits definitive conclusions. Cardiac assessment—including structure, function, electrical activity, and CK levels—showed no differences. Brain MRI revealed no structural or regional abnormalities, and BMD was similar across all sites. These negative findings consistently suggest that embryo splitting may not adversely affect heart, brain, or bone development, although the small sample size limits definitive conclusions.

Reproductive assessment, including gamete functionality, in vitro embryo development, and in vivo pregnancy, provided preliminary evidence of gamete functionality and embryonic developmental competence. Oocytes from cynomolgus sp-monkeys showed normal maturation, morphology, fertilization, blastocyst formation, and developmental timing. Similar trends were observed in rhesus sp-monkeys. Sperm motility and acrosomal integrity of cynomolgus sp-monkeys were also normal. Importantly, embryo transfer resulted in successful pregnancy and live births, providing direct evidence that embryos derived from cynomolgus sp-monkey gametes can establish pregnancy in surrogate females. This functional assessment provides the supportive evidence for the safety of embryo splitting, although it does not by itself confirm normal fertility in the sp-monkeys. To our knowledge, no previous study has systematically confirmed fertility in NHP offspring derived from this technique [18,19,20].

Embryo splitting may theoretically affect derived offspring through several mechanisms, including altered epigenetic reprogramming, genomic imprinting, placental development, and long-term health. In the present study, multi-system evaluation of adult cynomolgus and rhesus monkeys did not reveal obvious adverse effects in growth, metabolism, cardiovascular function, neurological development, or reproduction. However, the small sample size limits statistical power, and subtle or long-term risks cannot be excluded. Larger cohorts and longer follow-up are therefore needed to confirm the safety of embryo splitting.

Several limitations should be noted. First, the sample size was relatively modest (sp-monkeys *n* = 7, controls *n* = 7). Although a total of seven sp-monkeys were included, statistical analyses were performed only in cynomolgus monkeys. Second, while the animals had reached adulthood, they did not encompass the full aging trajectory. Therefore, late onset conditions such as metabolic syndrome, neoplasia, and age-related cognitive decline remain to be further evaluated. Additionally, brain structure in the sp-monkeys was assessed solely by MRI, and no more in-depth neurobehavioral analyses were conducted. Finally, we did not use embryos produced from both sp-monkey gametes and transfer them into female sp-monkeys as surrogates. Ideally, both gametes and the surrogate recipient should be from sp-monkeys to directly assess reproductive ability. Semen analysis was also limited by the small number of males, and semen volume, appearance, and detailed sperm morphology were not systematically recorded; therefore, only functional sperm parameters were assessed. Future studies are needed.

## 5. Conclusions

In summary, this multi-system evaluation of 6-year-old NHP offspring provides preliminary evidence that embryo splitting is compatible with normal growth and organ development and did not reveal obvious impairment of reproductive function in this cohort. Subtle glucose changes with preserved overall tolerance may reflect metabolic plasticity rather than pathology. However, these findings should be interpreted cautiously because of the small sample size, the availability of only two rhesus sp-monkeys, the inconsistent use of sp-monkey gametes, the absence of male rhesus sp-monkeys, and the use of surrogate females for pregnancy. The results therefore provide supportive rather than conclusive safety data; larger cohorts and longer follow-up are needed.

## Figures and Tables

**Table 1:** Hematological parameters values of sp-monkeys and controls.

	Cynomolgus Monkey		Rhesus Monkey	
Parameter (Unit)	Sp-Monkeys (*n* = 5)	Controls (*n* = 4)	Sp-Monkeys (*n* = 2)	Controls (*n* = 3)
RBC (10^12^/L)	6.19 ± 0.63	6.18 ± 0.47	5.41 ± 0.14	5.63 ± 0.09
HGB (g/dL)	15.14 ± 1.23	14.48 ± 0.51	13.4 ± 0.71	14.13 ± 0.22
HCT (%)	49.3 ± 4.18	48.10 ± 1.72	43.05 ± 1.34	44.75 ± 0.37
MCV (fL)	79.74 ± 1.68	78.10 ± 3.93	79.6 ± 0.42	79.55 ± 1.14
MCH (pg)	24.5 ± 0.91	23.50 ± 1.40	24.75 ± 0.64	25.13 ± 0.26
MCHC (g/L)	30.72 ± 0.60	30.10 ± 0.51	31.10 ± 0.71	31.58 ± 0.33
PLT (10^9^/L)	386 ± 149.77	399.75 ± 131.62	261 ± 101.82	333.5 ± 63.53
RDW-SD (fL)	39.2 ± 1.75	36.3 ± 3.11	40.2 ± 1.27	35.53 ± 2.53
RDW-CV (%)	13.78 ± 1.12	13.13 ± 0.46	13.85 ± 0.21	12.25 ± 0.69
PDW (fL)	11.63 ± 1.71 *	14.53 ± 1.68	13.4 ± 2.82	10.98 ± 1.41
MPV (fL)	10.76 ± 1.15	11.95 ± 0.64	11.65 ± 1.06	10.50 ± 1.21
P-LCR (%)	30.7 ± 10.63	40.10 ± 5.88	36.55 ± 8.98	26.98 ± 8.90
PCT (%)	0.41 ± 0.14	0.48 ± 0.14	0.31 ± 0.14	0.35 ± 0.03
NEUT# (10^9^/L)	3.58 ± 0.47	4.62 ± 2.63	2.63 ± 1.44	2.79 ± 0.99
LYMPH# (10^9^/L)	5.20 ± 1.33 **	8.85 ± 1.36	5.13 ± 2.52	5.91 ± 1.80
MONO# (10^9^/L)	0.81 ± 0.34	1.07 ± 0.15	0.51 ± 0.09	0.61 ± 0.24
EO# (10^9^/L)	0.23 ± 0.19	0.43 ± 0.19	0.08 ± 0.03	0.11 ± 0.03
BASO# (10^9^/L)	0.01 ± 0.00	0.03 ± 0.01	0.03 ± 0.01	0.03 ± 0.01
NEUT% (%)	36.84 ± 6.71	29.7 ± 13.39	32.95 ± 21.85	30.01 ± 9.37
LYMPH% (%)	52.26 ± 6.81	59.93 ± 11.87	59.75 ± 21.57	62.15 ± 8.32
MONO% (%)	8.54 ± 4.28	7.15 ± 0.85	6.05 ± 0.21	6.23 ± 1.44
EO% (%)	2.28 ± 1.58	3.05 ± 1.63	0.95 ± 0.21	1.33 ± 0.72
BASO% (%)	0.08 ± 0.04	0.18 ± 0.10	0.30 ± 0.14	0.28 ± 0.10
WBC (10^9^/L)	9.82 ± 1.21 *	15.00 ± 2.23	8.36 ± 1.20	9.44 ± 2.10

* *p* < 0.05; ** *p* < 0.01.

**Table 2:** Biochemical parameter values of sp-monkeys and controls.

	Cynomolgus Monkey		Rhesus Monkey	
Parameter (Unit)	Sp-Monkeys	Controls	Sp-Monkeys	Controls
AST (U/L)	42.38 ± 8.57	39.58 ± 5.31	29 ± 1.41	33.25 ± 4.64
ALT (U/L)	56.24 ± 25.19	62.9 ± 18.72	28 ± 1.41	40.25 ± 16.46
TBIL (μmol/L)	1.43 ± 0.28	1.18 ± 0.76	2.27 ± 1.45	3.15 ± 1.11
DBIL (μmol/L)	0.5 ± 0.24	0.30 ± 0.24	1.30 ± 0.85	2.20 ± 1.89
TP (g/L)	71.44 ± 4.86	72.33 ± 5.02	69.4 ± 1.41	72.98 ± 4.26
ALB (g/L)	43.36 ± 4.77	42.08 ± 2.31	46.9 ± 0.99	47.4 ± 0.62
GLB (g/L)	28.10 ± 1.32	30.25 ± 4.18	22.45 ± 2.33	25.58 ± 3.65
A/G	1.55 ± 0.19	1.41 ± 0.19	2.10 ± 0.27	1.88 ± 0.24
UREA (mmol/L)	7.47 ± 1.26	7.11 ± 2.32	6.24 ± 0.08	6.64 ± 0.34
CREA (μmol/L)	58.8 ± 9.54	57.8 ± 8.81	52.00 ± 4.24	58.00 ± 5.48
UA (μmol/L)	2.18 ± 1.22	3.93 ± 0.95	0.50 ± 0.71	1.00 ± 0.82
HDL-CH (mmol/L)	1.96 ± 0.35	2.09 ± 0.85	1.62 ± 0.44	2.02 ± 0.33
LDL-CH (mmol/L)	1.35 ± 0.53	1.21 ± 0.56	1.56 ± 0.18	1.76 ± 0.45
TG (mmol/L)	0.74 ± 0.57	0.48 ± 0.16	0.75 ± 0.34	0.56 ± 0.08
TC (mmol/L)	3.43 ± 0.86	3.51 ± 1.46	3.24 ± 0.64	3.84 ± 0.83
APOA1 (g/L)	1.40 ± 0.14	1.30 ± 0.36	1.26 ± 0.27	/
APOB (g/L)	0.32 ± 0.14	0.27 ± 0.15	0.40 ± 0.07	/
CK (U/L)	145.86 ± 46.10	134.85 ± 75.16	86.5 ± 3.54	154 ± 42.97
LDH (U/L)	492.00 ± 237.78	411.50 ± 165.64	219.00 ± 0.50	212.00 ± 44.52
Ca (mmol/L)	2.33 ± 0.13	2.30 ± 0.08	2.30 ± 0.01	2.47 ± 0.66
P (mmol/L)	1.76 ± 0.24	1.83 ± 0.37	1.76 ± 0.01	1.84 ± 0.45

## Data Availability

The original contributions presented in this study are included in the article/Supplementary Materials. Further inquiries can be directed to the corresponding author(s).

## Supplementary Materials

The following supporting information can be downloaded at: https://www.mdpi.com/article/10.3390/ani16213459/s1, Figure S1: Analysis of rhesus sp-monkeys body condition; Figure S2: Analysis of rhesus sp-monkeys cardiac structure and function; Figure S3: Analysis of oocytes and sperm in cynomolgus sp-monkeys; Supplementary Video S1: MRI scan videos of adult monkeys derived from sp-monkeys and control monkeys. Source date.

## Abbreviations

The following abbreviations are used in this manuscript: Sp-monkeys: monkeys derived from split embryos; BMD: Bone Mineral Density; NHPs: non-human primates; IVGTT: Intravenous glucose tolerance test; DXA: dual-energy X-ray absorptiometry; AoS: aortic sinus diameter; AA: ascending aortic diameter; LAD: left atrial diameter; RVD: right ventricular diameter; IVS: interventricular septal thickness; LVPW: left ventricular posterior wall thickness; LVID: left ventricular end-diastolic diameter; LVIDS: left ventricular end-systolic diameter; EDV: left ventricular end-diastolic volume; SV: stroke volume; EF: ejection fraction, FS: fractional shortening, RA-T: right atrial transverse diameter; RA-L: right atrial longitudinal diameter; AV: outflow velocity; VBM: gray matter volume; WMV: white matter volume.
