# Supplementary material for: Health Status of Adult Cynomolgus and Rhesus Monkeys Produced by Embryo Splitting
Source: Animals (Basel). 2026 Nov 2;16(21):3459. doi: 10.3390/ani16213459 (PMC13761992; doi:10.3390/ani16213459)
Supplement: Supplementary file 1 [file animals-16-03459-s001.zip › animals-4589445-supplementary.pdf]

Health status of adult monkeys produced by embryo splitting

Zifan Li[1,2,#], Yitong Yin[1,#], Yashi Wu[1], Yanchao Duan[2], Zhigang Chen[2], Yuandong Niu[2], Nannan Gong[2], Yu Kang[1,2], Yuyu Niu[1,2,3]*, Chenyang Si[1,2,*]

Correspondence: niuyy.lpbr.cn (Y.N), sicy@lpbr.cn (C.S)

The PDF file includes:
Figure S1 - 3

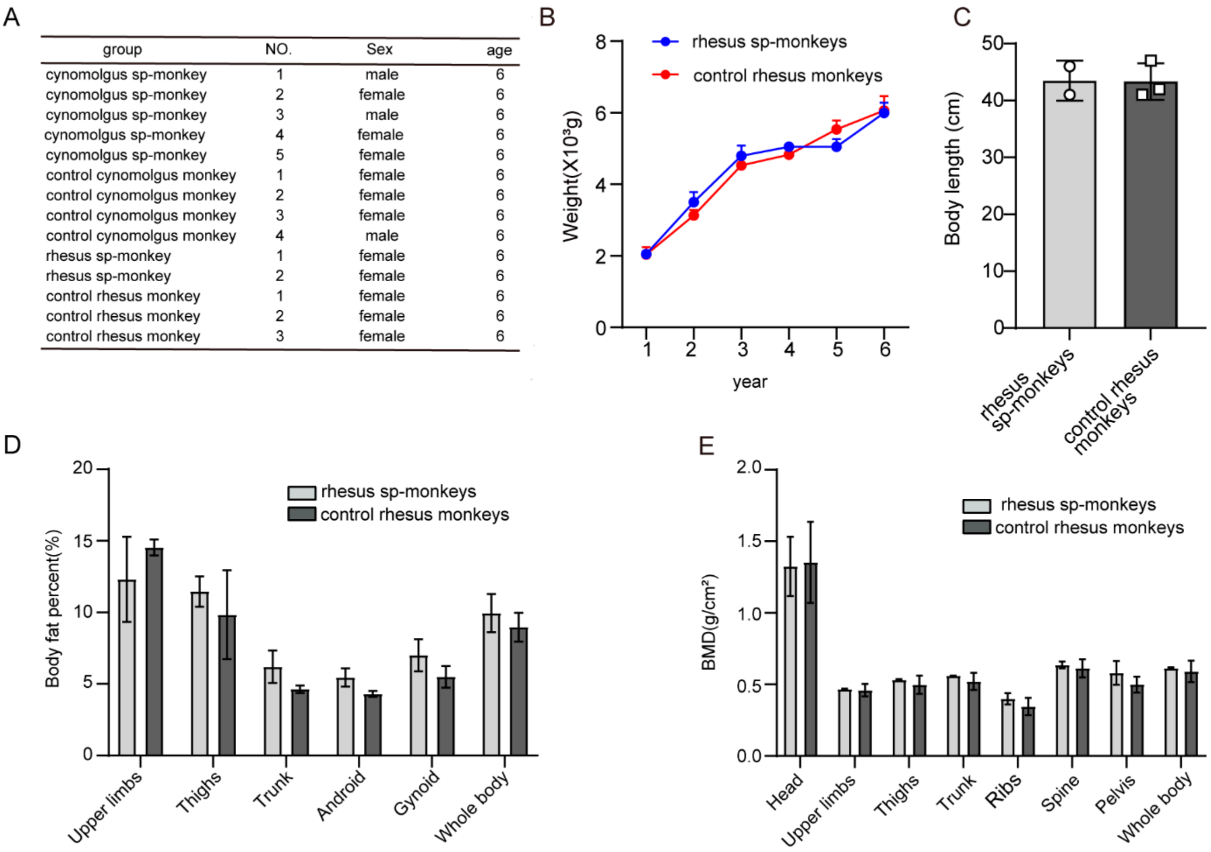

group	NO.	Sex	age
cynomolgus sp-monkey	1	male	6
cynomolgus sp-monkey	2	female	6
cynomolgus sp-monkey	3	male	6
cynomolgus sp-monkey	4	female	6
cynomolgus sp-monkey	5	female	6
control cynomolgus monkey	1	female	6
control cynomolgus monkey	2	female	6
control cynomolgus monkey	3	female	6
control cynomolgus monkey	4	male	6
rhesus sp-monkey	1	female	6
rhesus sp-monkey	2	female	6
control rhesus monkey	1	female	6
control rhesus monkey	2	female	6
control rhesus monkey	3	female	6

Figure S1. Analysis of rhesus sp-monkeys body condition

A. The table summarizes the animal information used in this study, including species, sex, and age.

B. Body weight changes from 1 to 6 years of age in rhesus sp-monkeys and controls.

C. The body length between rhesus sp-monkeys and controls.

D. Comparative quantitative analysis of body composition in the upper limbs, thighs, trunk, android fat region, gynoid fat region, and whole body of rhesus sp-monkeys and controls.

E. Comparisons of bone mineral density in the head, upper limbs, thighs, trunk, ribs, spine, pelvis, and whole body between rhesus sp-monkeys and controls.

All data are presented as mean ± SD. (rhesus sp-monkeys, n = 2; control rhesus monkeys, n = 3).

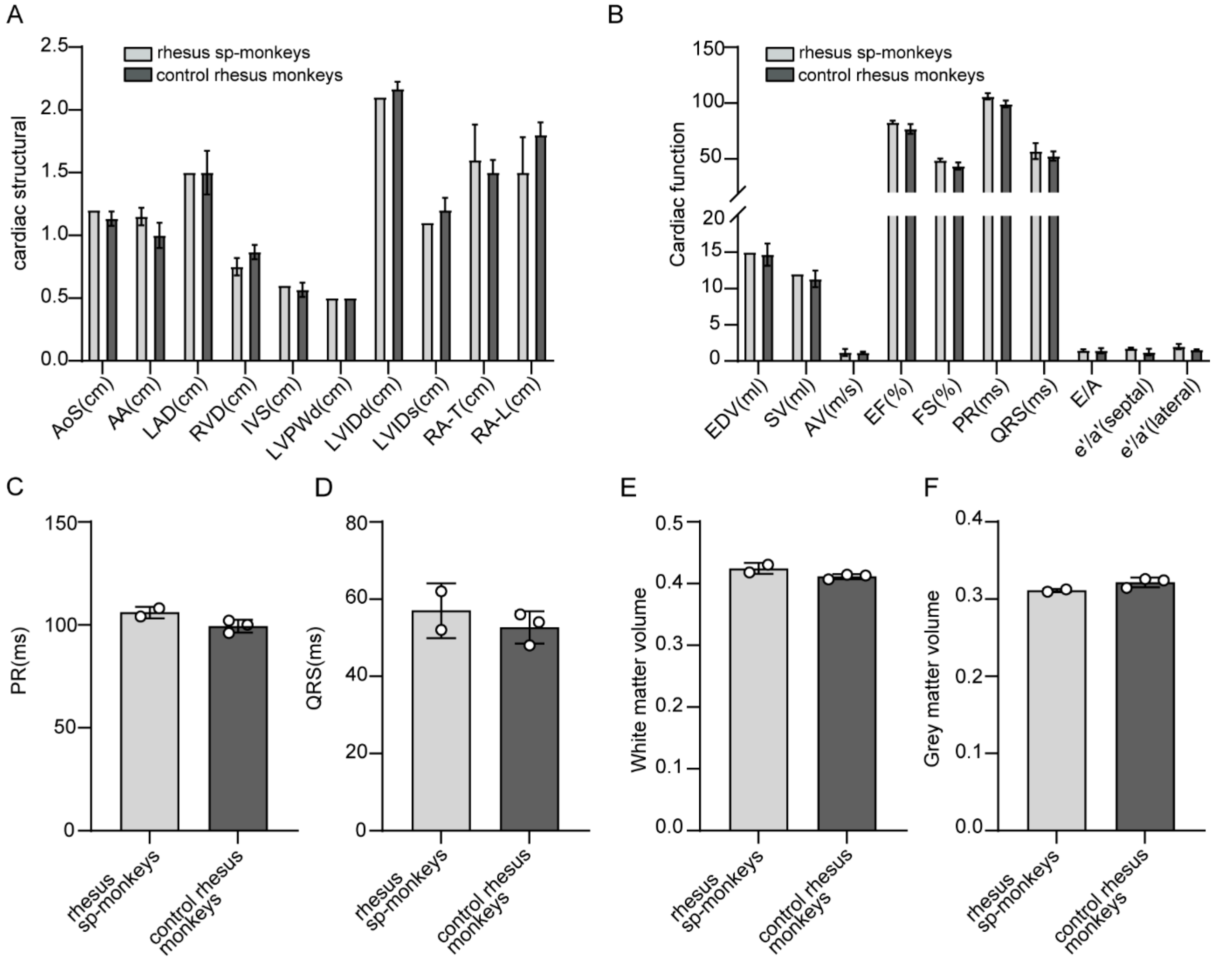

Figure S2. Analysis of rhesus sp-monkeys cardiac structure and function

A-B. Quantitative comparisons of cardiac structural parameters (A) and function (B) between rhesus sp-monkeys and control. AoS, aortic sinus diameter; AA, ascending aortic diameter; LAD, left atrial diameter; RVD, right ventricular diameter; IVS, interventricular septal thickness; LVPWd, left ventricular posterior wall thickness; LVIDd, left ventricular end-diastolic diameter; LVIDs, left ventricular end-systolic diameter; RA-T, right atrial transverse diameter; RA-L, right atrial longitudinal diameter. E/A, mitral inflow velocities E/A ratio; e′/a′ (septal), tissue Doppler imaging (TDI) mitral annular e′/a′ ratio at the septal annulus; e′/a′ (lateral), TDI mitral annular e′/a′ ratio at the lateral annulus; EDV, left ventricular end-diastolic volume; SV, stroke volume; EF, ejection fraction; FS, fractional shortening; AV, peak aortic valve outflow velocity.

C-D. Quantitative comparison of the PR interval (C) and QRS duration (D).

E-F. Atlas of white matter (E) and gray matter (F) regions constructed from T1- and T2-weighted images, with volumetric comparisons of corresponding regions.

All data are displayed as mean ± SD. (rhesus sp-monkeys, n = 2; control rhesus monkeys, n = 3).

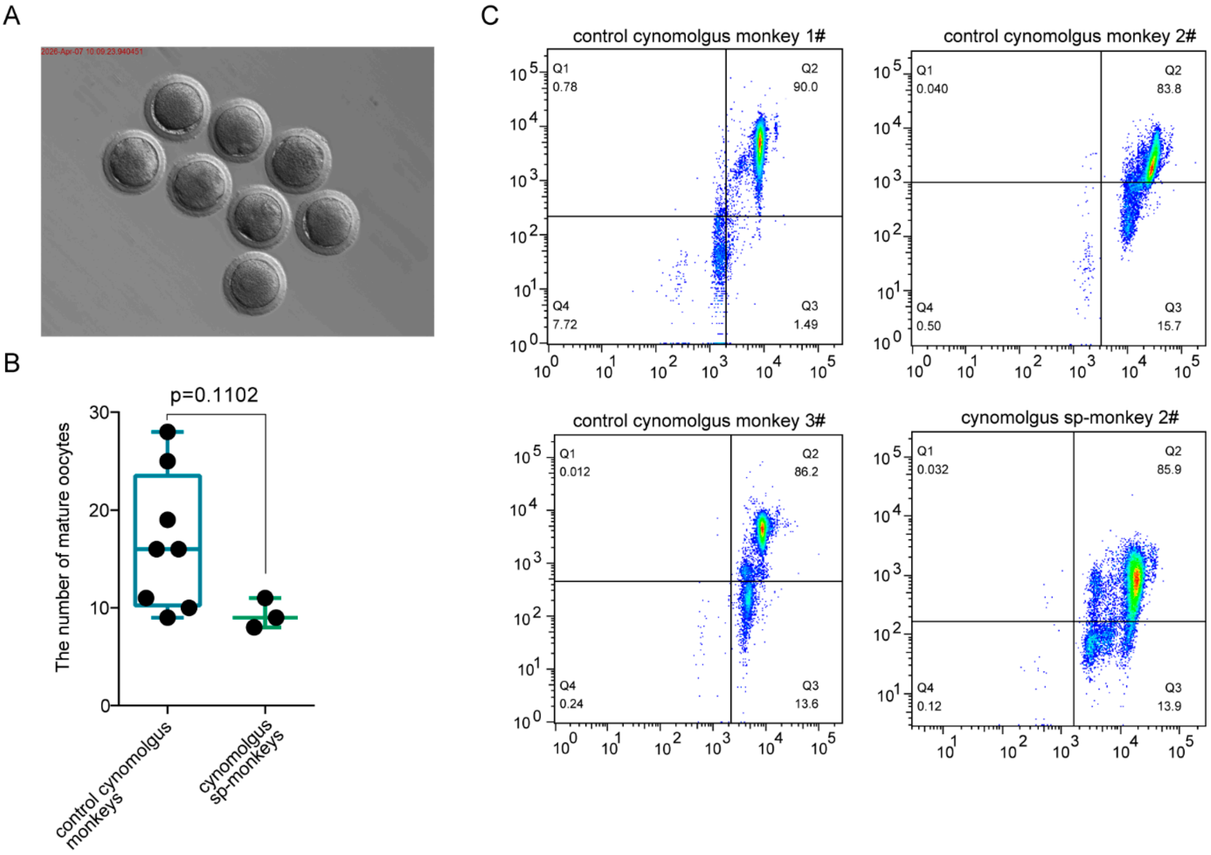

Figure S3. Analysis of oocytes and sperm in cynomolgus sp-monkeys

A. Representative bright-field images of oocytes from cynomolgus sp-monkeys.

B. Statistical analysis of the number of oocytes retrieved after superovulation in cynomolgus monkeys and control cynomolgus monkeys. Data are presented as mean ± SD; unpaired two-tailed Student's t-test. (controls, n = 8; sp-monkeys, n = 3).

C. Flow cytometric analysis of mitochondrial membrane potential in spermatozoa from cynomolgus sp-monkeys and control cynomolgus monkeys.
